# Improving Oral Health in Prisons (PriOH): Protocol for a Randomized Controlled Trial

**DOI:** 10.2196/60817

**Published:** 2024-12-11

**Authors:** Emilie Bryne, Kathrine Høyvik Bergum, William Gilje Gjedrem

**Affiliations:** 1 Oral Health Centre of Expertise Rogaland Stavanger Norway; 2 Faculty of Health and Social Sciences University of South-Eastern Norway Kongsberg Norway; 3 School of Business and Law University of Stavanger Stavanger Norway

**Keywords:** intervention, motivational interviewing, oral care, marginalized groups, correctional services, implementation, oral hygiene, oral health, randomized controlled trial, RCT, prison, dental care, pilot study, people living in prison

## Abstract

**Background:**

People living in prisons often experience poor oral health, which could be attributed to their limited access to (dental) care, financial constraints, and a general lack of awareness and prioritization toward their oral hygiene. A pilot study involving motivational interviewing (MI) has shown promising results for improving the oral health outcomes of people living in prisons.

**Objective:**

The protocol for this study aims to assess the efficacy of integrated MI and oral hygiene packages in improving oral health among people living in prisons, compared to controls without added MI.

**Methods:**

This oral health in prisons study is a multicenter, randomized, double-blinded controlled trial that recruited inmates from 4 prisons in Rogaland County. The trial aimed to recruit 320 participants before randomly allocating them to either a control or intervention group. The intervention group received MI, consisting of a 30-minute session encouraging inmates to discuss their current and desired oral health behaviors and attitudes, highlighting discrepancies to motivate change. Assessments were conducted at 4 and 12 weeks after initiation. The primary outcome measurement is the mucosal-plaque scores to assess oral health behaviors, attitudes, and oral hygiene. Secondary outcome measurements are oral hygiene routines, sugary food and drink intake, oral health perception, and oral health-related questions.

**Results:**

Data collection started in November 2021 and ended in June 2023. A total of 327 participants were recruited, of which 126 received the intervention.

**Conclusions:**

Integrating MI in oral health programs at prisons can significantly improve the oral health of incarcerated individuals. Should the results from this study demonstrate efficacy, it could be valuable insight for policy makers, oral health practitioners, and correctional services in addressing the needs of a traditionally underserved group before being scaled up to enhance dental care practices in prisons.

**Trial Registration:**

ClinicalTrials.gov NCT05695443; https://clinicaltrials.gov/study/NCT05695443

**International Registered Report Identifier (IRRID):**

DERR1-10.2196/60817

## Introduction

### Background

Poor oral health tends to affect people living in prisons disproportionately [1,2], which may be attributed to the lack of accessible oral health services within correctional facilities, financial barriers, a lack of awareness and priority, and limited use of oral health hygiene products and tools [3]. Scholars argue that ensuring good oral health for people who have been incarcerated and are in the process of reintegrating into society is particularly important because it can lead to a more successful outcome [4]. Assuring good oral health for people living in prisons is described as an international challenge. It is often related to the lack of staffing and logistical issues during transfer to and from dental clinics [3,5,6]. These barriers may also explain why, in Norwegian prisons, emergency dental treatment tends to be the most sought reason for dental care [6].

Another critical factor as to why people living in prisons tend to have poorer oral health is that they are more likely to have disadvantaged backgrounds, experienced social exclusion, and often have lifestyles increasing the risk of developing oral diseases [7-10]. With the prison parameters directly limiting one’s ability to engage in risk behaviors leading to poor health, one could argue for exploiting these contextual parameters and providing people living in prisons with oral health improvement measures.

Promoting and assuring good oral health for people in prisons has been advocated at structural levels by The World Health Organization’s Prison Programmes, calling for health promotion to be equal outside as within prisons [11], and at the dental practitioners’ level [6]. Assuring and promoting good oral health is different worldwide. In Norway, along with France and the United Kingdom, the health care for people living in prisons falls under the responsibility of the national public health department [8]. Moreover, Norwegian legislation states that public dental services should work preventatively toward those incarcerated. Currently, people living in prisons receive free dental care for acute matters from their first day of incarceration [12,13]. After that, regular dental examinations, with means to prevent and treat oral decay, are made accessible and free of cost when incarceration exceeds 3 months [13]

Nonetheless, a recent Norwegian regional report highlighted the need to strengthen the preventative and health-promotive measures [12]. In light of this recent report and the international literature revealing a lack of interventions despite this population scoring disproportionally poorer on oral health measures [14], the Oral Health Centre of Expertise Rogaland developed an intervention that included motivational interviewing (MI), with means to promote good oral health behaviors and thereby prevent oral diseases for people living in prisons, and results from a pilot study on this reveals promising findings [15].

MI is a collaborative, person-centered technique that strives to achieve change through conversational techniques [16]. The driving conversation aims to last around 30 minutes, exploring the participant’s current and desired (oral) health behaviors and attitudes. An MI-led conversation assumes that asking questions illuminating the discrepancy between the desired and current state and behavior motivates the person to change [16]. Although MI leading to behavior changes has been well studied [17], more evidence is needed to uncover whether this technique effectively leads to oral health-related changes within prison parameters. This protocol outlines a randomized control trial, assessing whether an MI-led conversation can enhance people living in prisons’ oral health, cleaning routine behaviors, and oral health attitudes.

From here on, this protocol refers to the MI-led conversation as the intervention. More details on the specific techniques of the MI-led conversation used in this study are outlined in the Methods section.

### Aims and Objective

Previous research and a pilot study conducted by the Oral Health Centre of Expertise Rogaland have shown that MI techniques can promote oral health and prevent oral health challenges by altering unhealthy habits and behaviors [15,16,18,19]. Our study is a randomized controlled trial that builds on the pilot study [15], to assess whether a conversation following MI techniques (the intervention) can enhance people living in prisons’ oral health, cleaning routine behaviors, and oral health attitudes. To answer this study’s aim, five hypotheses have been outlined:

H1: The intervention will reduce observed plaque and gingival inflammation for prisoners (mucosal-plaque score [MPS]).

H2: The intervention will lead to improved reported oral hygiene routines, particularly the brushing of teeth.

H3: The intervention will lead to a reduced intake of food and drinks that contain sugar, specifically in between meals.

H4: The intervention will lead to improved perceptions about their oral health. It improves how they view their oral health, they consider their oral health as more important, and they report an increased wish to improve it and have increased efforts to improve it.

H5: The intervention will lead to more questions asked during the clinical examination, suggesting increased curiosity concerning their oral health.

## Methods

### Design

A multicenter randomized controlled trial with a parallel group design and superiority framework will be used to assess the effectiveness of the intervention in improving oral health behaviors, attitudes, and hygiene for people living in prisons. From November 2021 to June 2023, all people convicted of serving time in any of the 4 correctional facilities (prisons) in Rogaland County and who passed a security and language assessment were recruited.

After giving oral consent, this study’s participants were randomly assigned to an intervention or augmented control group that was blocked at the prison level. This means, although study participants were randomized, there was an equal number of participants in both the intervention and augmented control groups within each of the 4 participating prisons (striving for 1:1 allocation). The randomization was computer-generated [20].

To prevent selection bias, this study’s concealment methods were computer-generated randomization at the back office by the research team. This, in practice, meant the research team received unidentifiable participant numbers that were added to the randomization pool. Prison staff were then notified which participant number should receive the intervention. They generated a list of participants, grouping them according to criteria, before giving this list to the dental practitioners who performed the intervention. This study did not use a data monitoring committee as the intervention was assessed as minimally invasive, alluding to a low risk of harming the participants.

**Figure 1:**
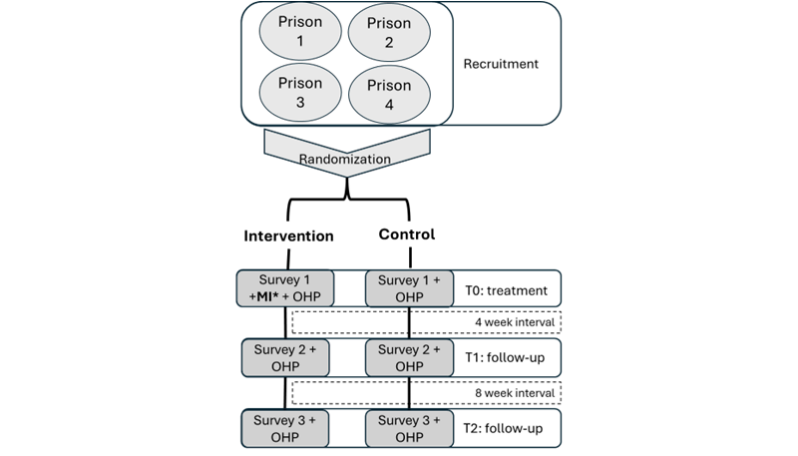
Study timeline and process. Surveys 1, 2, and 3 included a clinical examination and questions related to their incarceration and oral hygiene routines, sugary food and drink intake, oral health perception, and mental health (MHI-5). Still, they varied in the length of their additional questions. *Dental practitioners performing the MI were blinded until they had finished the survey (consisting of a clinical examination and questionnaires). Thus, although the research team had already randomized participants into intervention or control groups, dental practitioners did not know the effects of the randomization until measurement scores were taken, to avoid bias. MHI-5: Mental Health Inventory-5; MI: motivational interviewing; OHP: oral hygiene package.

A 2-fold survey, consisting first of a clinical examination before a questionnaire, was administered at 3 data collection points that aimed at a 4- and 8-week interval (T0, T1, and T2 in Figure 1). At the end of each survey, all participants were given an oral hygiene package (OHP) consisting of mouthwash, toothpaste, a toothbrush, and interdental cleaners (plackers and soft gum picks). Providing all participants with the same material to enhance their oral health also ensured that all participants had equal equipment and opportunity at baseline to enhance their oral health. Moreover, ethical considerations also played a role in giving the control group an OHP, which is detailed in the Ethical Considerations section. Given the prophylactic effect the OHP could have and the presumably increased focus on oral health related to the assessment, the control group is an augmented control group (hereon defined as the control group), as it does not reflect a regular prison context.

### Study Setting, Participants, and Procedures

This study has already collected data. Thus, procedures related to recruitment are written in the past tense. Participants were recruited from 4 prisons in Rogaland, 3 being high-security and 1 being low-security.

Study recruitment was a process that varied due to logistics in the prisons and constant enrollment. Recruiting participants who were already serving time in prison was done by a dental staff or prison officer approaching eligible inmates (Table 1) and informing them about this study. For new inmates arriving at the prisons, recruitment became embedded on their first day of entry, because they were informed about study participation by prison staff while learning about the prison’s procedures and drills. Participants gave their preliminary consent to participate within a few days after being informed about this study by notifying prison staff. To ensure voluntary motivation for study participants, recruiting staff were sensitive in their approach and study participants were reminded throughout that participation was voluntary and that they could withdraw at any point without repercussions.

**Table 1:** Inclusion and exclusion criteria for study participation.

	Inclusion	Exclusion	Rationale	Procedure
Language	Norwegian speakers	English^a^ speakers and non-Norwegian speakers	This study lacked trained and calibrated staff to conduct the motivational interview in languages other than Norwegian	Prison staff assessed this either at face value or by asking a standard question from an official Norwegian language test that detects a person’s ability to engage in a simple conversation
Security	Those who were not at risk of harming themselves or others	Inmates whose incarceration was in isolation	This study did not seek to compromise any of the staff or inmates’ security	Prison staff assessed this throughout this study’s period
Age	≥18 years	—^b^	The prison facilities in Norway are split for adults and adolescents. This study recruited from adult prisons	By recruiting at the given prisons, all prisoners aged older than 18 years were included
Gender	All genders: male, female, and other	None	—	By recruiting at the given prisons, a female division at one prison was a natural part of the participation pool

^a^Considering the potential number of English speakers, the survey and consent form was outlined and administered in English to capture more baseline data. These will not be included in analyses assessing the effects of motivational interviewing.

^b^Not applicable.

Study participants were then randomized to either the intervention or control group. Formal written consent was given to the dental practitioners after they had been randomized (T0, Figure 1), and prison staff was not present, minimizing the superiority of the observer, which could be linked to the prison staff. Information about study participation was similar across all 4 prisons; they received verbal and written information, which was repeated by the dental staff before the written consent was obtained. At the recruitment stage, individuals were screened for language and study eligibility criteria, including inmates who could speak English or Norwegian and who passed a security assessment (Table 1). Only Norwegian-speaking participants were allocated to either the control or intervention group based on the assumption that dental practitioners would struggle with MI in a different language. Table 1 outlines this study’s inclusion and exclusion criteria.

As outlined in Figure 1, this study has 3 data collection points: baseline (T0) and follow-up (T1 and T2). At T0, the participants were administered survey 1, which was 2-fold and consisted of a clinical examination and questionnaires. The clinical examination assessed the mean number of Decayed, Missing, and Filled Permanent Teeth [20] and the MPS [21]. The questionnaire in survey 1 consisted of 83 questions on their incarceration, oral hygiene routines, sugary food and drink intake, oral health perception, and oral health-related questions on dental anxiety (Modified Dental Anxiety Scale) [22,23] and quality of life (Oral Health Impact Profile-14) [24,25], sociodemographic and socioeconomic factors, use of dental and health services, general health and mental health (Mental Health Inventory-5 [MHI-5]) [26] and alcohol and drug use (Alcohol Use Disorders Identification Test–Consumption and Drug Use Disorders Identification Test–Consumption) [27,28]. Survey 1 was expected to take around 60 minutes. Data collection with participants receiving MI was expected to last around 90 minutes, where an additional 30 minutes were allocated to the intervention.

The follow-up phases, T1 and T2, each lasted around 30 minutes. At T1, survey 2 was administered, which contained the MPS oral examination and questions related to their incarceration, oral hygiene routines, sugary food and drinks intake, oral health perception, the MHI-5, and a short screening for learning disabilities (Hayes Ability Screening Index) [29,30]. At T2, survey 3 was administered, which contained the MPS oral examination and questions related to their incarceration, oral hygiene routines, sugary food and drink intake, oral health perception, and the MHI-5.

### MI and Training

The dental practitioners who led the MI conversation were employed by public dental services (Rogaland, Norway) and consisted of 6 dental hygienists, 1 dentist, and 3 dental assistants. After reading about this study in an email sent to all public dental practitioners in Rogaland County, these dental practitioners applied for positions as research participants. Following the application was a short interview between the research group and dental practitioner that assessed their motivation and outlined the details of participation.

Within prison parameters, these dental practitioners performed the MI-led conversation with incarcerated people (Figure 1), closely following techniques described by Miller and Rollnick [16]. The MI-led conversation involved engaging, focusing, evoking, and planning by asking open-ended questions, and conversing affirmative, reflective, and summarizing with this study’s participants. In practice, the typical MI-led conversation would start with the dental practitioner asking openly, “What cleaning routines do you have for your teeth?” or “What benefits could you get from brushing your teeth twice daily?” Following the respondent’s answer to that open question would often lead to an affirmation by the dental practitioner, “You are truly trying hard to take good care of your teeth” or “You have managed to have good cleaning routines multiple times in the past.” By using the reflection technique, during the MI-led conversation, the dental practitioners would typically repeat or use synonyms, extracting the underlying opinion or gut feeling, or dually highlighting the participant’s pros and cons. The MI-led conversation closes with summarizing the participants’ words.

By using open-ended questions and techniques such as affirmation, reflection, and summarizing, the conversation is steered from first concentrating on the task of being engaged with the interviewee, toward becoming more focused on illuminating and clarifying the goal and desired behavior changes, to then evoking the individuals’ strength and motivation. The MI-led conversation ends with planning how this change is to come about and does so by incorporating a SMART (small, measurable, attainable, realistic, and time-specific) plan embedded in the action plan. The dental practitioner and participant jointly outline this action plan that summarizes what and how the change would come about. The action plan was a handout that this study’s participants could keep, hang in their room, and serve as a reminder of the participants’ goals and steps in their change process, and help them overcome obstacles. The action plan and aid dental practitioners had in the MI-led conversation are uploaded as Multimedia Appendix 1.

Before conducting the MI-led conversation, the dental practitioners underwent a comprehensive three-day training seminar led by an MI-certified specialist from the Regional Alcohol and Drug Competence Center in Rogaland County (KORUS). This training, which included group work, discussions, and a practice session was designed to equip them with the necessary skills and knowledge. The dental practitioner’s conversations were also recorded, transcribed, and graded by KORUS in Bergen, with direct feedback provided. The MI specialist at the Center for Alcohol and Drug Research at Stavanger University Hospital was also available for continuous guidance if needed, ensuring that the dental practitioners were well-prepared for their role.

### Measurements

#### Primary Outcome

The primary outcome measurement to assess the interventions’ effectiveness in improving oral health behaviors, attitudes, and oral hygiene for people living in prisons is the MPS. This score is a composite measure that includes assessments of mucosal inflammation (MS) and plaque, which are indicators of changes in dental hygiene routines and attitudes [21]. The MPS was assessed at three time points: baseline (T0), midpoint (T1), and at the completion of the study (T2). The assessment procedure is simple and minimally invasive, ensuring minimal participant discomfort. Both the MS and plaque scores range from 1 to 4.

During scoring, dental practitioners undertaking the clinical MPS assessment were advised to score lower (a score of 1) in cases where there is doubt between the score of 1 and 2, and higher (a score of 4) for cases where there is doubt between the score of 3 or 4—combined the MS and plaque give an MPS score that has a total score of 8 and a minimum score of 2. Assessment guidelines define a score of 2-4 as good or acceptable, 5-6 as not acceptable, and 7-8 as in no way acceptable [21].

#### Secondary Outcome

The secondary outcome measurements this study is interested in are oral hygiene routines, sugary food and drink intake, oral health perception, and oral health-related questions, which were assessed in the survey at T0, T1, and T2.

For oral hygiene routines, participants were asked about their use of dental hygiene products such as toothbrushes, toothpaste, mouthwash, floss, and toothpicks over the last 4 weeks.

Participants were asked about their consumption within the last 4 weeks for sugary food and drink intake.

For oral health perception, participants were asked about their view of their health, the importance of good oral health, and their willingness to improve and take better care of their oral health. Dental practitioners noted how often study participants asked about their oral health during the clinical examination.

Participants were also asked about using the action plan (handed out after the MI-led conversation, Multimedia Appendix 1), which also serves as a secondary outcome measure.

Lastly, the survey collected descriptive data from participants that will be used to describe the population based on sociodemographic and socioeconomic factors, the use of dental health services and health services, and indicators of general and mental health (MHI-5). Additionally, the burden of oral decay (Decayed, Missing, and Filled Permanent Teeth), substance use patterns (assessed by Alcohol Use Disorders Identification Test–Consumption for alcohol and Drug Use Disorders Identification Test–Consumption for drug use), the presence of learning disabilities (Hayes Ability Screening Index), levels of dental anxiety (Modified Dental Anxiety Scale), and the impact of oral health-related quality of life (Oral Health Impact Profile-14) were also collected and will be used as descriptive data for the population. These data will also serve as covariates in the statistical analysis evaluating variables associated with oral health.

### Sample Size and Justifications

Determining the needed sample size and power was assumed before data collection (ClinicalTrials.gov NCT05695443). Setting the α at .05, power of 0.80, for a nonparametric Wilcoxon-Mann-Whitney *U*-test. Data from previous studies using MI to improve oral health deviates from this study’s population and in scoring plaque. Studies with a plaque index score as the primary variable to explain the effect reported a plaque range from 0.286 to 1.213 [31,32]. A conservative calculation through the computation tool G*Power (Heinrich-Heine-Universität Düsseldorf), determined a sample size of 320 people living in prisons to meet an observable effect size of 0.80, which was strived for. Given data has already been collected, we have performed a sensitivity power analysis through the computation tool G*Power, to compute the required effect size with our sample size of 126 in the intervention group and 157 in the control group. Setting the same parameters as prior (α at .05 and power at 0.8) gives us an effect size of 0.305.

### Data Analysis

Analysis will be performed using the statistical software package SPSS (IBM Corp). The unit of analysis is at the individual level. As this study is interested in uncovering the group differences between the intervention and control groups, a nonparametric Wilcoxon-Mann-Whitney *U*-test will be run, and group means will be compared at T0, T1, and T2 (Figure 1). The outcome of interest (primary outcome variable) is whether the participants’ MPS scores have changed since baseline.

The main covariate is the preintervention (T0) MPS index score.

### Double Blinding

Study participants and dental practitioners were blinded to group assignments during the initial oral examinations and questionnaires (survey 1) at baseline (T0). This double blinding was maintained until the dental practitioners completed the initial survey, after which they conferred with a list to check if participants were in the intervention or control group. At the follow-up data collection points (T1 and T2), dental practitioners might have recalled the participants’ group assignments, potentially introducing bias. To mitigate this, our study incorporated MPS assessments from 2 independent dental practitioners at these stages, and scores were confidentially maintained to prevent bias.

As a final measure to ensure objectivity, data analysis will be conducted by a researcher who was not involved in any previous stages of this study, effectively establishing a third level of blinding during the analytical stage.

### Missing Values

Missingness is presumed to be unrelated to the observed and unobserved data, thus, as missing completely at random. For analytical purposes, variables missing in the primary outcome will be handled with an intention-to-treat (ITT) analysis approach, allowing this study to use baseline data.

### Ethical Considerations

The Norwegian Regional Committee for Medical and Health Research Ethics (282231), Criminal Services, and the Norwegian Agency for Shared Services in Education and Research (300281) have approved this study.

This study relies on informed written consent. To ensure prison inmates have been fully informed about study participation, recruitment processes have relied on help from both dental practitioners and prison staff. The written consent form was read out loud as well as handed out to ensure those with reading deficiencies understood what study participation entailed. As English speakers were included for baseline data, a consent form in English was also presented. Participants’ ability to opt-out was stated at the recruitment phase as well as reminded of during data collection. All collected data has been deidentified.

Ethical deliberations led this study to administer the questionnaire part of the surveys verbally to ensure participants fully comprehended the questions asked. Verbally performing the questionnaires also gave latitude for the participant to ask questions in return and the dental practitioners to remind participants that they could withdraw or refrain from answering at any point without any form of consequence.

All participants (intervention and control group and English speakers) received an OHP because of the assumption that not all prison inmates had equal and sufficient tools to perform and potentially enhance their oral hygiene routines. Considering participants were randomly allocated into the control or intervention group, it was considered unethical to only give a hygiene package to those enrolled in the intervention group. The OHP passed prison security clearance.

All study participants (intervention and control group and English speakers) also received a clinical examination, which allowed dental practitioners to refer participants who needed dental treatment. This referral line was established due to ethical concerns, deeming it unethical for dental practitioners to detect oral diseases needing treatment without attending to them.

This study chose not to ask and collect data on what offenses were committed due to ethical and safety concerns. It was believed that knowing the crime offended could affect the dental practitioner’s approach and conduct toward this study’s participants.

Although this study was assessed as minimally invasive for its participants, all studies can impose a risk of impairment resulting from participation. To ensure this study limited its adverse events, dental practitioners who conducted the intervention and survey had direct contact with prison staff and were on alert, reacting to any signs of harm following the survey or intervention.

## Results

Data collection started in November 2021 and finished in June 2023. Out of the total 327 participants, 283 were given the questionnaire in Norwegian, while 44 received it in English. A total of 126 of these received the intervention. This study is still plotting all data and will refrain from analysis until this protocol has been reviewed. All results from the data collection phases are expected to be ready by 2026.

## Discussion

### Principal Considerations

The global prison population has grown by 27% since 2000, and currently, over 11 million people are living in prisons [33]. Being incarcerated directly limits the ability to access health care services. This might be attributed to the lower outcome measurements of oral health for inmates in prisons. Moreover, routine interventions in prison contexts are scarce [14], and there is a lack of focus on oral health promotion in prison research [34].

This calls for a change in how oral health is promoted for people living in prisons. The opportunities the MI techniques provide could lead to more health promotion, less need for acute treatment, saving resources for the prison, and reducing the number of prison inmates who need transport to the dental clinic. This study was developed to improve the oral health of people living in prisons. Thus, the findings from this study will allow us to understand if the currently underserved population could benefit from an alternative approach.

Moreover, providing MI to the prison population can serve as a low-cost, effective procedure for increasing prison inmates’ motivation to routinely care for their oral health by engaging a dental hygienist to conduct the conversation. Cost-effective studies could investigate whether MI with people living in prisons could be a more cost-effective way to build a dental clinic at the prison site.

### Strengths and Limitations

A strength of this study is ensuring that dental practitioners who were undertaking the clinical examination were calibrated and received the same MI training. Dental practitioners were calibrated through a one-day workshop where they met physically to discuss clinical oral examinations, questionnaires, and procedures. Fifteen clinical photos were scored for MPS: First, the photos were individually scored before being jointly discussed and agreed upon. As the dental practitioners varied in background, the 3 participating dental assistants received an additional hour of calibration, compensating for their lack of clinical experience. This supplementary calibration hour for the dental assistants involved reviewing and MPS scoring 50 clinical photos. Lastly, 2 independent MPS scores on the participants were retained to avoid biases during data collection by having 2 dental practitioners scoring the same person individually. Data obtained from the calibration, describing the interrater variability between the 2 independent MPS scorers, will be analyzed and published along with the main study.

Although dental practitioners who led the intervention were trained, they could also lack confidence in the therapeutic role due to the novelty of using MI in prisons, which could affect the intervention delivery and outcome.

A notable limitation of this study is that both groups received an OHP comprising oral hygiene products that could act as a preventative for oral decay. Moreover, survey 1 included 83 questions, which were asked before the MI-led conversation. These questions might have caused fatigue in participants, deterring the effects of the MI.

Lastly, this study deviates from the pilot study by including the MI communication element of *reflection* [15,16].

### Conclusion and Directions for Dissemination

This study aims to test and measure the effects of an MI-led conversation on improving people living in prisons’ oral hygiene, cleaning routines, and attitudes. Granted that the intervention has an effect, the results can aid in how public dental health services can promote oral health for an underserved population, thereby increasing their objective of acting health promotive.

Results from this study will be published in national and international academic journals. This study’s findings will also be shared through conferences for academics and practitioners and various forms of public media. A report of the project will also be written and shared with the collaborating partners and will also be open to the public.

This protocol has followed SPIRIT (Standard Protocol Items for Randomized Trials) guidelines.

## Appendix

Multimedia Appendix 1 The action plan and aid dental practitioners had in the MI-led conversation. MI: motivational interviewing.

## Abbreviations

ITT: intention-to-treat
KORUS: Regional Alcohol and Drug Competence Center in Rogaland County
MHI-5: Mental Health Inventory-5
MI: motivational interview
MPS: mucosal-plaque score
MS: mucosal inflammation
OHP: oral hygiene package
SMART: small, measurable, attainable, realistic, and time-specific
SPIRIT: Standard Protocol Items for Randomized Trials

## Data Availability

Data collected from this study are sensitive, and participants have not consented to freely distributing it, limiting its availability.
