# Supplementary material for: Improving Oral Health in Prisons (PriOH): Protocol for a Randomized Controlled Trial
Source: JMIR Res Protoc. 2024 Dec 11;13:e60817. doi: 10.2196/60817 (PMC11669880; doi:10.2196/60817)
Supplement: Multimedia Appendix 1 [file resprot_v13i1e60817_app1.pdf]

Motivational Intervieweing

REMEMBER TO:

CREATE DISCREPANCY: focus on the disadvantages of the current situation

SOLVING AMBIVALENCE: focus on the benefits of change

ASK FOR PERMISSION TO GIVE ADVICE

EXPLORE-OFFER-EXPLORE

DANCE

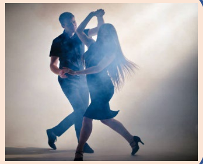

The Communication Skills:

Ask open-ended questions:

-Tell us more about...
-What routines do you have for ...
-What benefits will it have for you...
- What would it be like for you to....

reflect:

-**Simple reflection**: use the same word (parrot) or synonym
-**Complex reflection**: underlying meaning or feeling (what does your own gut feeling tell you?)
-**Two-sided reflection**: sheds light on both sides of the issue (both the advantages and the disadvantages), ending with the positive. Use "at the same time as" rather than "but"

summarize:

--You have mentioned several things...
To sum up...
-So you.....and you....

Affirm:

-You're really trying...
-You have a lot of thoughts about this
- You have managed this several times before

Action Plan

Action Plan

REMEMBER

1. THE INMATE'S PERSPECTIVE: THE PLAN MUST BE BASED ON THE INMATE'S IDEAS AND THOUGHTS EXPRESSED IN THE MI CONVERSATION

2. DEFINE SPECIFIC BEHAVIOR: IT MUST BE SOMETHING THE INMATE WILL DO (E.G. BRUSH THEIR TEETH TWICE A DAY)

3. THE PLAN MUST HAVE A DIRECTION: START WITH SOMETHING, STOP SOMETHING, CONTINUE WITH SOMETHING, INCREASE, DECREASE, MORE OFTEN/LESS OFTEN

USE: OPEN-ENDED QUESTIONS – AFFIRMATION – REFLECTION – SUMMARY

ASK FOR PERMISSION!

EXPLORE-OFFER-EXPLORE!

THINK SMART:

- Specific
- Measurable
- Attainable
- Realistic
- Time-limited

Action Plan

- What changes do you want to make in terms of your oral health?
- What is the main reason why you want to make a change
- When do you want to start?
- What will it take for you to follow your plan?
- What could be potential obstacles or difficulties in achieving your goal?
- How do you solve the obstacles?
